# Dramatic therapeutic play in nursing care for children and adolescents’ mental health: theoretical considerations

**DOI:** 10.1590/0034-7167-2024-0664

**Published:** 2025-12-08

**Authors:** Giulia Delfini, Circéa Amália Ribeiro, Edmara Bazoni Soares Maia, Vanessa Pellegrino Toledo, Ana Paula Rigon Francischetti Garcia

**Affiliations:** I Universidade Estadual de Campinas. Campinas, São Paulo, Brazil; II Universidade Federal de São Paulo. São Paulo, São Paulo, Brazil

**Keywords:** Nursing, Mental Health, Child, Adolescent, Play and Playthings., Enfermería, Salud Mental, Niño, Adolescente, Juego e Implementos de Juego.

## Abstract

**Objectives:**

to develop a theoretical proposal on the nursing care process for children and adolescents with mental health problems, considering the structuring of the subject of the unconscious to establish the nurse-patient relationship through dramatic therapeutic play.

**Methods:**

qualitative theoretical study, with a theoretical-methodological framework, developed from psychoanalysis.

**Results:**

to offer care developed in the nurse-patient relationship through the nursing process, nurses can consider playing as a means of communication for children in psychological distress, anchored in the theoretical support of playing in psychoanalysis to carry out dramatic therapeutic play sessions, permeating the “Establishing a relationship”, “Exploring”, “Playing” and “Closing the session” stages, considering their respective theoretical supports, the nursing process phases, and nurses’ attitudes and actions.

**Final Considerations:**

the study presents a possibility of care for children and adolescents with psychological distress, developed in the nurse-patient relationship during the dramatic therapeutic play stages.

## RESUMEN

**Objetivos:**

desarrollar una propuesta teórica sobre el proceso de atención de enfermería a niños y adolescentes con problemas de salud mental, considerando la estructuración del sujeto inconsciente para establecer la relación enfermera-paciente a través del juego dramático terapéutico.

**Métodos:**

estudio teórico cualitativo, con un marco teórico-metodológico desarrollado a partir del psicoanálisis.

**Resultados:**

para ofrecer cuidados en la relación enfermera-paciente a través del proceso de enfermería, la enfermera puede considerar el juego como un medio de comunicación para el niño con sufrimiento psicológico, anclado en el sustento teórico del juego en psicoanálisis para realizar sesiones de juego terapéutico dramático, permeando las etapas “Estableciendo relación”, “Explorando”, “Jugando” y “Finalizando la sesión”, considerando sus respectivos sustentos teóricos, las fases del proceso de enfermería, y las actitudes y acciones de la enfermera.

**Consideraciones Finales:**

el estudio presenta una posibilidad de cuidado a niños y adolescentes con sufrimiento psicológico, desarrollada en la relación enfermera-paciente durante las etapas del juego dramático terapéutico.

## INTRODUCTION

Mental healthcare for children and adolescents, a population that covers the age range from 0 to 19 years old^(1)^, is a global public health challenge, given that 25% to 30% of children and adolescents suffer from mental disorders^(2)^. In Brazil, it is estimated that 13% of these people suffer from psychological distress, and access to community services for care can be complex, in addition to lacking continuity^(3,4)^.

The biopsychosocial care model considers not only biological and social factors, but also lived experiences, as elements that influence health and illness, giving importance to subjects’ subjectivity and mental health in their life context^(5)^. In this model, psychodynamics with psychoanalytic influence is a comprehensive part of child psychiatry, when considering other forms of treatment in addition to psychotropic drugs and behavioral therapies^(6)^.

In this scenario, psychoanalysis emerges as a well-defined therapeutic field supported by the technique of analysis through play^(6,7)^. For psychoanalyst Ricardo Rodulfo, there is nothing significant about how a child is structured that does not involve playing. In addition to being the most important means of their expression, play is also important because, even though they lack verbalized words due to their lack of language mastery, they can still convey their questions and anxieties through play^(7)^.

Considering nursing care, we can mention play therapy, a psychiatric technique used by healthcare professionals to treat children with mental health problems^(8)^. When considering the private care of nurses, play constitutes a form of approach to be used in pediatric care to ensure comprehensive care for children^(8,9)^. In this context, therapeutic play (TP) is considered soft care technology designed by nurses concerned about children’s distress during hospitalization, and are supported by Resolution 546/2017^(8)^.

TP is a play designed to help children alleviate anxiety caused by atypical experiences, and is classified into three categories: dramatic or cathartic, instructional, and physiological function enabler^(8,10)^. Dramatic therapeutic play (DTP) allows children to release emotions, relieve anxiety, and express feelings, fantasies, and desires, as well as helping to build a therapeutic relationship with children and their families^(11,12)^. This is developed in four complementary and interdependent stages. Each stage can occur at the beginning, middle, or end of treatment. They can also occur simultaneously in a single session, namely: Establishing a bond; Exploring; Dramatizing; and Stopping playing^(12)^.

DTP is the most appropriate TP modality in children’s mental health because it allows discourse to emerge. In other words, communication with nurses will be established through play^(7-9,12)^. Nursing has historically faced barriers to developing care for children with mental health problems, such as a mismatch between training and practice and alienation from the knowledge of other professionals^(13)^. To address these issues, nurses must adopt a theoretical framework that supports the therapeutic relationship and allows for autonomous, transformative care^(14)^.

In this context, when working with children in psychological distress, DTP emerges as an alternative for exclusive care by nurses, and to read what is presented by children in the sessions, it is essential to adopt a theoretical framework^(8,14)^. Psychoanalysis emerges as a possibility for reading play during DTP, by taking it as a structured language like the unconscious, characterized by significant articulation^(9)^. In other words, it is necessary to consider that the unconscious is structured as a language and that this occurs through signifiers^(7,9)^. Thus, play, in addition to being a language for accessing the unconscious, is also a significant practice that has toys as its object^(7,9)^.

To systematize the implementation of DTP as an intervention, the option is taken to use the nursing process (NP) as a possible care strategy that favors nurses taking an autonomous position as a therapeutic agent in the nurse-patient relationship^(13,14)^.

Thus, this study is justified by the increasing rates of psychological distress in the child and adolescent population and the high demand for intensive care, with community services being difficult to access and segmented; by the scarcity of studies that address the process of nurses caring for children with psychological distress, which may demonstrate a knowledge gap in nursing care, including with regard to the use of DTP; and by the importance of nurses being involved in this care, anchored in a theoretical perspective for the implementation of assistance^(2,4,8,9,13)^.

## OBJECTIVES

To develop a theoretical proposal on the nursing care process for children and adolescents with mental health problems, considering the structuring of the unconscious subject to establish the nurse-patient relationship through DTP.

## METHODS

This article is a product of the master’s dissertation “*Cuidado à criança com transtorno mental por meio do Brinquedo Terapêutico Dramático: abordagem pela relação intersubjetiva*”^(15)^, deposited in the institutional repository of the public university where it was defended, with access via the link: https://hdl.handle.net/20.500.12733/9773.

### Theoretical-methodological framework

Psychoanalysis, operating within the field of language, was used as a theoretical and methodological framework, in which the function of speech in transference, understood as the repetition of the forms of satisfaction used to establish relationships, is examined through an analysis of transferential repetition, which reveals the language of the unconscious as expressed in the signifying logic^(16,17)^.

Therefore, working with signifiers that are inserted into language and conceptualized as phonemic units is a way of considering the unconscious. These signifiers allow a word to acquire multiple meanings since they are unconscious manifestations that must obey structural aspects characterized by repetition, significant gravitation, and direction. Significant gravitation occurs when a word assumes a singular meaning for subjects beyond the meaning agreed upon in common language. This favors a new outline for the subjective position. Direction determines subjects’ preferred paths for relating to others^(7,18)^. It is worth noting that a signifier does not have a single meaning but rather carries invaluable effects of signification^(7,18,19)^.

The knowledge of the unconscious is incomplete, and in this work, this gap was characterized by the nursing team’s difficulty establishing their role and functions in caring for children with psychological distress. This resulted in unfounded care^(13)^.

DTP was takes as a theoretical framework for developing the care process^(12)^. To align with the psychoanalysis theoretical framework, the concept of the unconscious was adapted from the works of Freud^(20)^, Lacan^(18)^, and Ricardo Rodulfo^(7)^. Subjects perceive their existence and name their psychological distress through language; however, children lack mastery of this in the context of childhood^(7,17)^. Playing emerges as their form of communication, as it allows them to express themselves and, consequently, access the unconscious^(7)^.

Although some authors argue that there is a difference between play and playthings, it is important to point out that, in Brazilian Portuguese, “*brincar*”, “*brincadeira*” and “*brinquedo*” may or may not be synonyms: while the first two always refer to the act of playing, the third can refer to both the action and the object^(8)^. In this context, when we refer to TP, we are talking about playing. Toys are a facilitator of play. Children do not stop playing because of a lack of toys. When toys are unavailable, children use their imagination to create and play^(8,12)^.

Adopting the psychoanalytic framework requires making theoretical changes and adjustments to the DTP stages so that subjects emerge from the unconscious^(17,18,20)^. Thus, the DTP session^(12)^ stages were transposed to the NP stages, being worked on in the following manner: Establishing a relationship; Exploring; Playing; and Closing the session.

### Study design

This is qualitative theoretical research, which consists of building connections and relationships between concepts derived from previous theories to develop a new study^(21)^.

### Methodological procedures

The work was divided into three stages. The first stage consisted of an integrative review of nursing care provided to children with mental health problems. This review enabled the understanding of significant concepts (which are repeated) about this care: healthcare models, family, diagnosis, and management.

The second stage aimed to understand the theoretical framework of playing as a language of the unconscious for reading the significant articulation^(7)^, which allowed, through uniformly suspended reading, to assimilate the psychoanalytic concepts of child, signifier, family myth, preliminary interviews, and playing.

Finally, the third and final stage was carried out through the articulation between the significant concepts of nursing care for children in psychological distress and the theoretical framework of playing as a language of the unconscious for reading significant articulation^(7)^, so that healthcare models were articulated to children and signifier; family to the family myths; diagnosis to preliminary interviews; and management to play.

### Data analysis

The data were analyzed according to the transference relationship between the researcher and the investigated content, insofar as the readings affected them beyond the rationality adopted in the study of the texts^(22,23)^. This occurs because reading to understand a theoretical articulation involves not only rationality, but also unconscious processes^(22,23)^.

The techniques of free association and uniformly suspended attention were employed. These techniques allow researchers to remain alert to new ideas by avoiding a categorical stance that directs their attention only to ideas that confirm what is already established^(22-24)^. Therefore, the researchers made an effort not to restrict the theory, allowing them to discover a non-totalizing conceptual development^(23)^.

## RESULTS

The results are presented in four charts that include each stage of DTP, its respective theoretical support (necessary concepts for developing the stages), NP phases, and nurses’ attitudes and actions (Chart 1, Chart 2, Chart 3 e Chart 4). It is worth noting that the charts contain universal theoretical guidelines that should be considered in each case in an attempt to provide individualized nursing care and recognize what each child needs at each stage of NP.

**Chart 1:** Theoretical support, nursing process phases, and nurses’ attitudes and actions in the “Establishing a relationship” stage of dramatic therapeutic play

Dramatic therapeutic play stage	Establishing a relationship			
**Theoretical support**	Biopsychosocial care model: comprehensive care with respect for uniqueness	Children’s record		Signifiers
	Free association	Transference		Family myth
	Request for healing addressed to professionals	Hysterization: transforming the symptom into an enigma		Family-centered care: including parents in treatment
	Preliminary interviews: transference and symptom functions		Addressing children: is there distress?	
**Nursing process phases**	Nursing assessment			
**Nurses’ actions and attitudes**	Consider children’s record, associated with the constitution of the subject of the unconscious so that care can take place taking into account the singularity, which enables the comprehensiveness advocated in the biopsychosocial care model	Introduce themselves, invite them to play and agree on the principles that will shape the sessions (day, time, duration and location)		Allowing themselves to express oneself subjectively beyond the position occupied in the family myth, discovering if there is distress
	Provide free play and “anything goes” for the emergence of free association	Include parents in treatment and listen to them in order to discover the main features of the family myth and the place occupied by children		Establish the transference relationship in preliminary interviews, allowing the addressing of a demand to be transformed into a symptom and this into an enigma

**Chart 2:** Theoretical support, nursing process phases, and nurses’ actions and attitudes in the “Exploring” stage of dramatic therapeutic play

Dramatic therapeutic play stage	Exploring		
**Theoretical support**	Dramatic therapeutic play toy box	Family myth: the Other as maternal body and treasure of signifiers	Preliminary interviews: transference, symptomatic and diagnostic functions
	Significant repetition	Acting out	Entry into treatment
	Play spontaneity	Oedipus complex	Make holes
	Children’s fate: phallus, symptom or ghost		
**Nursing process phases**	Nursing assessment	Nursing diagnosis	Nursing planning
**Nurses’ actions and attitudes**	Allow exploration of toys and repetition of play so that the signifiers inherited from the Other are enacted as a family myth	Recognize signifiers to enable the encounter of new ones or to reestablish their relationship with them, unblocking a chain of signifiers	Identify whether there is spontaneity in play and encourage the recovery of their playful ability so that there is more than just family desire
	Act out together with parents to identify family myths, the place occupied by children in the family and in the desire of Other	Allow holes to be made, losing respect for the toy as a condition for being	Transform the symptom into an enigma and draw hypotheses about the structural diagnosis
	Allowing play as an acting out mediator of care		

**Chart 3:** Theoretical support, nursing process phases, and nurses’ actions and attitudes in the “Playing” stage of dramatic therapeutic play

Dramatic therapeutic play stage	Playing		
**Theoretical support**	Playing as a means of communication	Acquisition of the notion of volume: container/content	Object and language relationship: “*fort/da*”
	Transference	Reciprocal inclusions	Weaning
	Formation of continuous surfaces	Symbolization of disappearance	Age to participate in dramatic therapeutic play
	Libidinal constitution and body structuring		
**Nursing process phases**	Nursing assessment	Nursing diagnosis	Nursing planning
	Nursing implementation		
**Nurses’ actions and attitudes**	Use transference to establish communication through play	Provide constancy of the setting and allow the formation of continuity with the environment so that it is inscribed in the form of a surface and can perform inside/outside operations	Enable games that allow symbolizing the disappearance of the Other so that separation does not become synonymous with bodily destruction
	Consider the elements that are attached as essential for the constitution of subjects in their libidinal corporality	Receive container/content games so that they can create a three-dimensional tube and acquire the notion of volume	Apply dramatic therapeutic play as a logical operator to help establish the functions of play, contributing to psychological constitution and body structuring
	Identify which logical difficulties children try to resolve through play and provide resources so that they can achieve the psychological development that is lagging behind		

**Chart 4:** Theoretical support, nursing process phases, and nurses’ actions and attitudes in the “Closing the session” stage of dramatic therapeutic play

Dramatic therapeutic play stage	Closing the session	
**Theoretical support**	Analytical setting	Logical time
	Subjectivity	Setting constancy
**Nursing process phases**	Nursing evolution	
**Nurses’ actions and attitudes**	Establish a continuity contract respecting singularity, considering the particularity of each case and enabling the emergence of the unconscious	Maintain the consistency of the setting by respecting the contract regarding the day, time, duration and location of the dramatic therapeutic play sessions, which helps in the construction of continuous surfaces
	Consider children’s reactions to the end of play and create interventions that help them process the separation	Consider the logical time and be aware that play can last for more than one session

## DISCUSSION

Using the psychoanalytic theoretical framework to adopt play as an intervention allows nurses to provide continuous care based on the therapeutic nurse-patient relationship during DTP. This enables the development of NP stages^(7,12,13)^. To this end, nurses must consider the stages of DTP development, as well as the theoretical support, attitudes, and actions that professionals should use.

### Establishing a relationship

At this stage, nurses use transference as one of the functions of preliminary interviews because it allows them to understand the logic of the unconscious^(16,25)^. The transference relationship begins with a personal introduction, an invitation to play, and the establishment of principles that will guide the DTP session, taking subjectivity into account^(12,17)^. When introducing themselves, children position nurses according to transference, which is a result of repeating the forms of satisfaction used when forming new relationships. This process is closely related to experiences in early childhood^(16)^.

Through the transference relationship, nurses adopt an attitude of applying the symptomatic function of preliminary interviews. That is, children make a demand that is answered with a symptom causing psychological distress. This demand must be transformed into a question, understood as a process of hysterization^(7,25,26)^. Thus, playing under a transference relationship enables nurses to access children’s unconsciousness, allowing the signifiers that shaped their psychological makeup to emerge during sessions^(27)^.

Free association also arises through transference, which is considered free play^(24,27,28)^. Nurses establish communication with children through play, since children do not yet have full command of language. Through play, children address a request for healing to professionals^(7,8)^. Allowing play as a means of communication is important because the function of speech under transference brings the reality of the unconscious into action within the scope of language^(7-9,12,16,17)^.

Therefore, it is important that play follows the principles of free association, as it should be spontaneous, without being directed, providing freedom of choice, the expression of feelings and the emergence of subjectivity^(7,8,27)^. Nurses should take the action of offering toys without direction, and should also not direct play, participating in it only when requested^(8)^. Free play is important because children bring meaning to it, making them the only ones able to create new possibilities for their lives^(8,29)^. Another characteristic of free play is that “anything goes”^(7)^. In other words, children play when they take any object, which is a theoretical formulation of the transition from the accidental to the necessary and structural. This is because they compose their bodily ego with this type of material^(7)^.

Through the transference relationship, nurses can develop hypotheses about the role of children in the family narrative, which is composed of a variety of signifiers and serves as a foundation for future relationships^(3,7)^. In order to understand the significant structure of children and their families, it is important for nurses to listen to parents, which allows recognizing the repeated signifiers in their speech^(9,27)^.

This listening can be punctual or continuous, with or without children present, but it is important to note that the inclusion and effective participation of family members in DTP sessions can only occur if the children request and/or authorize it^(7,8)^. However, it is important to receive the discourse of parents/family members at the level of children’s treatment, allowing them to express themselves subjectively and identify any distress^(27)^. In this way, they are given the opportunity to establish their own subjective position under transference, ceasing to be passive objects of parental ghosts and taking an active role in their own desires^(27)^.

This approach enables nurses to consider children as subjects of the unconscious, taking into account their psychological distress and unique circumstances, which allows for the comprehensive care adopted in the biopsychosocial care model^(5,7,27)^. To this end, it is necessary to consider children’s records, which are associated with the constitution of subjects of the unconscious^(18,20,27)^. Therefore, in the first stage of DTP, nurses act as therapeutic agents and perform nursing assessments.

### Exploring

At this stage, a toy box is created and given to children so that they can explore it freely^(12,30)^. It is suggested that it contain: everyday objects, such as plastic food, miniature furniture and cars; representative figures, such as dolls (simulating family members, healthcare professionals, animals); materials that stimulate creativity, such as colored pencils, paper and modeling clay; and toys that allow the expression of feelings, such as pacifiers, baby bottles, weapons, tanks and soldiers^(8,30)^. It is when they come into contact with this box, made up of a variety of toys, that exploration takes place^(12)^.

Nurses need to pay attention to one of the main functions of play as a therapeutic practice: that it is reproduced incessantly, which configures a significant repetition^(7,31)^. This insistence must be received, because what is repeated tends to symbolic elaboration by the repetition movement itself, which must be read as a knot to be deciphered^(7,9,31)^.

Such repetition paves the way for acting out when children are unable to remember what they have repressed due to resistance mechanisms, presenting it through action^(31-33)^. Nurses must recognize this repetition and, when they do, question children in the transference relationship to help them express themselves through words instead of actions^(17,31-33)^.

Play repetition is related to the concept of a signifier. When children stage the appropriation of Others’ signifiers, they indicate their position in the family myth and their place in the desire of the Other. This can manifest as phallus (marked by desire); symptom (attached to a certain trait of the family construction in which it is impossible to inscribe a difference); or ghost (when the paternal function is erased)^(7)^.

Furthermore, it enables professionals to formulate hypotheses about children’s role in language by examining the denial mechanisms of the Oedipus complex. This complex addresses the triangular relationships between parents and children and can evoke intense emotions such as hatred and love towards parental figures^(7,18,34)^. As a result, it becomes possible to identify the structures of neurosis, psychosis, or perversion. This enables nurses to perform the diagnostic function of preliminary interviews^(7,25,27)^. The transference and symptomatic functions initiated in the previous stage are also present here and signal the start of treatment^(7,17,25)^.

Children’s symptoms may be a response to family problems. It is necessary to continue the inclusion of parents in treatment, as begun in the previous stage. At birth, babies need an interpreter, or a maternal function, who will name their actions. As they interpret their state and seek meanings to appease it, they are inserted into the logic of language, represented by the signifiers offered to them^(9,35)^. In other words, this Other can be understood as a maternal figure and a treasure trove of signifiers^(7)^. Thus, it is essential to identify the signifiers of the family myth, delimit each family member’s position, and recognize if children are affected by the new and different^(3,7,27)^.

As a result, during DTP sessions, children either seek signifiers that represent them, express a desire to transform existing signifiers, or more frequently, seek to eliminate some of them^(7)^. In this case, nurses facilitate conditions so children can find or reestablish their relationship with a signifier.

At this stage, it is also necessary to determine whether children are playing spontaneously or to satisfy perceived demands from adults^(7)^. If children are unable to play spontaneously, nurses must help them recover or develop their playful abilities^(36)^.

The final attitude observed at this stage is the allowance of play involving the creation of holes, as producing a hole is a prerequisite for existence^(7)^. The most intense and regular activities in the first year of life are associated with producing holes in the body of the Other. This is a moment when the baby removes and appropriates signifiers to constitute itself as a desiring subject, thereby separating and differentiating itself from the body of the Other^(7,37)^. In this context, nurses must allow children to treat toys as tools for digging holes and removing signifiers^(7)^.

Thus, in the “Exploring” stage, nurses continue assessment, prepare diagnoses and begin nursing planning.

### Playing

At this stage, children play in a way that allows them to communicate with nurses. They create imaginary situations that allow them to express and cope with their daily lives, anxieties, fears, tensions, and conflicts. It is a way for them to verbalize their feelings and life experiences^(7,12)^.

The primary function of play, especially during the first year of life, is the libidinal constitution and structuring of the body. In other words, play resembles the process of extracting materials from the body of the Other to create one’s own^(7,37)^. Children use these materials sequentially, extracting and manufacturing continuous surfaces^(7)^.

Thus, constant encounters with perforable Other allow babies to achieve integration, i.e., they find the raw material necessary for constructing continuous surfaces^(7)^. It is essential for children to inscribe themselves as surfaces so they can perform inside/outside operations^(7)^.

Pediatrics recommends creating routines for babies, which are nothing more than creating surfaces: the Other must provide the means to build a daily routine through them^(7)^. It is important for nurses to develop DTP in a protected environment with a consistent setting, away from excessive external disturbances, to provide the tranquility necessary for building surfaces^(7)^.

The second function of play relates to the second stage of body structuring, during which a baby engages in games involving containers and contents^(7)^. In this context, it is important to consider the concept of reciprocal inclusions. In other words, the relationship between container and content is reversible and ambiguous. It is a mistake to assume that internal content is different from an external container^(7)^.

From forming continuous surfaces and elaborating a two-dimensional tube, the notion of volume gradually emerges, allowing differentiation between internal/external and subject/object. This makes it a three-dimensional tube^(7)^. In this context, nurses adopt an attitude of accepting play associated with the container/content in DTP sessions^(7)^. For instance, they engage in repetitive games of taking objects out and putting them inside a box or opening and closing cabinets and drawers in a disorganized way.

The third function of play appears around 9 months, and manifests itself through hide-and-seek games, practices of disappearing and appearing^(7,20,31)^, resulting in more elaborate games in which the enjoyment of hiding is essential^(7)^.

Disappearance, which until then had not caused any pleasure or anguish, becomes a libidinal event, and the child finds it funny and asks for repetition^(7)^. One of the functions attributable to play, known as “*fort/da*”, is therefore to symbolize disappearance and give representation to absence^(7,20,31)^. In this scenario, the absence of the gaze of the Other no longer fractures the continuous surfaces, and children experience existence without it^(7,31)^.

This way of playing is associated with weaning, particularly from the maternal gaze^(7)^. These brief moments when children let go of the supportive gaze allow for the dual enjoyment of hiding and reuniting^(7)^. Thus, in the multiple games of DTP sessions, children must ask themselves: how can something that is absent and not visible exist?

The success of an operation depends on the success of the previous one. In other words, damage to the body’s surface will interfere with the “*fort/da*” operation because separation will be equated with self-destruction^(7)^.

For this reason, one of the innovations of this study is the suggestion to expand the age range for using DTP. Until now, DTP has been recommended for children aged 3 to 12 years^(8)^. The study proposes expanding its use to children and young adults by considering play as a symbolic device that enables the emergence of discourse and, consequently, access to the unconscious. This idea is supported by the psychoanalysis theoretical framework^(7,16,18)^. Nurses must consider applying DTP as a logical operator to assist in implementing the functions that playing has for psychic constitutions. Its use should not be restricted to a specific age group because psychological distress can lead to precociousness or delay in subjects’ psychological development^(7,27)^.

In the first few months of life, it is possible to observe children responding to anguish somatically, i.e., with their bodies^(7)^. In this context, DTP can be applied at the beginning of the symbolic structuring of language, around six months of age. Through logical reading and case-by-case assessment by nurses, it can be extended until subjects, whether children or adolescents, need to organize continuous surfaces, acquire the notion of volume, and resignify separations. Failure to develop these functions can have repercussions beyond the age of 12^(7,16)^.

It is important for nurses to consider that children of DTP application age may manifest symptoms and needs that reveal failed structural play functions. In these cases, sessions will include repetitive plays that demonstrate developmental deficits^(7)^. Based on this, professionals must provide the necessary resources during sessions so children can achieve their psychic development that is lagging behind, considering, for this purpose, the evaluation of the logic of its constitution.

To achieve this objective, professionals adopt an attitude of accepting children’s plays. Based on what is expressed, they ask themselves: what logical difficulties do children have to face at certain times, and how do they try to solve these difficulties through play? Based on this, nurses offer care that allows children to process the issues they present through the NP implementation stage.

It is important to consider that, as this stage may reveal new discoveries about children and their symptoms, it is also possible for nurses to formulate other nursing diagnoses and think about new interventions to be implemented. This moment indicates the close interconnection between the various stages of NP.

### Closing the session

Finally, at this stage, the psychoanalytic technique of the contract with patients was considered. This contract establishes the analytical setting and defines the length, frequency, and form of payment of the sessions^(25)^. Above all, this contract should be considered in the context of ethics rather than rules. In other words, it should be drawn up as a psychoanalytic act, in which professionals acknowledge the unconscious and provide treatment tailored to each case^(25)^.

Therefore, DTP session contract must include conditions rather than imposed rules. It is also necessary to consider logical time as opposed to chronological time since respect is not for the clock but for significant relationship^(38,39)^. In this way, the contract is specific to each child, providing consistency in the setting while taking into account nurses’ observations of children’s needs.

Details such as the times and frequency of sessions must be agreed upon according to the particulars of each case. Nurses must establish conditions so that the DTP session contract benefits the children. Moreover, nurses should observe how children react at this time. Do they accept the end of the play? Do they start playing when they realize the session is over? Or do they try to prolong it, indicating that the play is not over for them yet^(12)^?

These reactions should be interpreted by professionals to adapt the length of each session to children’s subjectivity. Nurses use an agreement that specifies the start and end times of the session. However, they can adjust the session’s duration as needed, recognizing that termination is also an action.

It is worth noting that the end of a session does not necessarily mean the end of play. In the psychoanalytic framework, logical time is considered^(38)^. Children play as if there were no interval between sessions, treating each session as a continuation of the previous one, and they decide when it will end^(12)^.

At this stage, when considering NP, nurses will perform nursing evolution and registration. In other words, they will assess how children respond to the implemented interventions and continue care or devise new, more appropriate treatment^(13)^ strategies, which should be recorded in the medical record.

### Study limitations

This study is limited by the difficulty of transmitting robust psychoanalytic theoretical concepts to nurses who choose to adopt them to develop care for children and adolescents with mental health issues. Psychoanalytic training occurs through personal analysis, supervision, and theory study^(40)^. The proposal defended here supports two of these pillars. In addition to providing care for children with mental health issues, applying it with supervision and studying the relevant concepts alongside personal analysis constitutes psychoanalytic training.

### Contributions to Nursing

This study presents a significant advancement in nursing for child and adolescent mental health: the possibility of continuous care developed through the nurse-patient relationship during the implementation of DTP stages. To develop these skills, nurses must be grounded in theoretical support for care. Here, play and its functions in psychological constitution are considered. From this foundation, nurses can provide care guided by NP stages.

## FINAL CONSIDERATIONS

This study aimed to develop a theoretical proposal for the nursing care process of children and adolescents with mental health issues through DTP. It considers the structuring of the unconscious subject to establish the nurse-patient relationship.

To this end, the proposed nursing care process considers play as a means of communication for children to develop the therapeutic relationship during DTP stages: Establishing a relationship; Exploring; Playing; and Closing the session. The care provided in NP stages occurs as nurses rely on theoretical support to inform their actions and attitudes towards children in psychological distress.

Bt theoretically supporting actions and attitudes for the development of NP, nurses who adopt the psychoanalytic theoretical framework can apply DTP as a logical operator to identify and act on failures in the constitution of continuous surfaces, the acquisition of the notion of volume, and the resignification of separations. This can assist in psychological development, sustaining subjectivity, and structuring a body that leads to an autonomous and singular experience.

## Data Availability

The research data are available within the article.
