# Supplementary material for: Dramatic therapeutic play in nursing care for children and adolescents’ mental health: theoretical considerations
Source: Rev Bras Enferm. 2025 Dec 8;78(6):e20240664. doi: 10.1590/0034-7167-2024-0664 (PMC12707536; doi:10.1590/0034-7167-2024-0664)
Supplement: Translation

# Translation: Brinquedo terapêutico dramático no cuidado de enfermagem em saúde mental infantojuvenil: considerações teóricas

**DOI:** 10.1590/0034-7167-2024-0664pt

**Authors:** Giulia Delfini, Circéa Amália Ribeiro, Edmara Bazoni Soares Maia, Vanessa Pellegrino Toledo, Ana Paula Rigon Francischetti Garcia

**Affiliations:** I Universidade Estadual de Campinas. Campinas, São Paulo, Brasil; II Universidade Federal de São Paulo. São Paulo, São Paulo, Brasil

**Keywords:** Enfermagem, Saúde Mental, Criança, Adolescente, Jogos e Brinquedos.

## Abstract

**Objetivos:**

desenvolver uma proposta teórica sobre o processo de cuidado de enfermagem ao público infantojuvenil em sofrimento psíquico, considerando a estruturação do sujeito do inconsciente para o estabelecimento da relação enfermeiro-paciente por meio do brinquedo terapêutico dramático.

**Métodos:**

estudo teórico qualitativo, com referencial teórico-metodológico, desenvolvido a partir da psicanálise.

**Resultados:**

para oferecer cuidado desenvolvido na relação enfermeiro-paciente por meio do processo de enfermagem, o enfermeiro pode considerar o brincar como meio de comunicação da criança em sofrimento psíquico, ancorando-se no suporte teórico do brincar em psicanálise para realizar sessões de brinquedo terapêutico dramático, permeando as etapas “Estabelecendo relação”, “Explorando”, “Brincando” e “Encerrando a sessão”, considerando seus respectivos suportes teóricos, as fases do processo de enfermagem, e as atitudes e ações do enfermeiro.

**Considerações Finais:**

o estudo apresenta uma possibilidade de cuidado ao público infantojuvenil em sofrimento psíquico, elaborado na relação enfermeiro-paciente durante a realização das etapas do brinquedo terapêutico dramático.

## INTRODUÇÃO

A assistência em saúde mental infantojuvenil, população que abrange a faixa etária dos 0 aos 19 anos de idade^(1)^, fundamenta um desafio de saúde pública global, visto que de 25% a 30% das crianças e adolescentes sofrem de transtornos mentais^(2)^. No Brasil, estima-se que 13% desses apresentam sofrimento psíquico, e o acesso a serviços comunitários para atendimento pode se mostrar complexo, além de carecerem de continuidade^(3,4)^.

O modelo de atenção biopsicossocial considera não só fatores biológicos e sociais, mas também as experiências vividas, como elementos de influência à saúde e doença, dando importância à subjetividade e saúde mental do sujeito em seu contexto de vida^(5)^. Neste modelo, a psicodinâmica com influência psicanalítica é parte integrante da psiquiatria infantil, ao considerar outras formas de tratamento além dos psicofármacos e das terapias comportamentais^(6)^.

Nesse cenário, a psicanálise surge como campo terapêutico bem delimitado sustentado pela técnica de análise por meio do brincar^(6,7)^. Para o psicanalista Ricardo Rodulfo, não há nada significativo na estruturação de uma criança que não passe pelo brincar, além de ser o meio mais importante de sua expressão, pois ainda que falte a ela as palavras verbalizadas por ainda não ter domínio da linguagem, é por meio dele que ela consegue transmitir suas questões e angústias^(7)^.

Considerando o cuidado de enfermagem, podemos citar a ludoterapia, técnica psiquiátrica utilizada por profissionais de saúde para o tratamento de crianças em sofrimento psíquico^(8)^. Ao considerarmos o cuidado privativo do enfermeiro, o brincar constitui uma forma de abordagem a ser utilizado na assistência pediátrica para garantir o cuidado integral à criança^(8,9)^. Neste contexto, o brinquedo terapêutico (BT) é considerado uma tecnologia leve de cuidado idealizada por enfermeiros preocupados com o sofrimento da criança durante a hospitalização, sendo respaldado pela Resolução nº 546/2017^(8)^.

O BT é um brinquedo estruturado para que a criança atenue a ansiedade causada por experiências atípicas, sendo classificado em três modalidades: dramático ou catártico, instrucional e capacitador de funções fisiológicas^(8,10)^. O brinquedo terapêutico dramático (BTD) permite à criança a descarga emocional, o alívio da ansiedade, e a expressão de sentimentos, fantasias e desejos, além de auxiliar na construção da relação terapêutica com a criança e sua família^(11,12)^. Este é desenvolvido em quatro etapas complementares e interdependentes, podendo cada uma prevalecer no início, meio ou final do tratamento, assim como acontecerem concomitantemente em uma única sessão, sendo elas: Estabelecendo vínculo; Explorando; Dramatizando; e Parando de brincar^(12)^.

O BTD configura-se como modalidade de BT mais adequada na saúde mental infantil, pois permite a emersão do discurso, ou seja, será por meio do brincar que se estabelecerá uma comunicação com o enfermeiro^(7-9,12)^. Historicamente, a enfermagem enfrenta barreiras para desenvolver o cuidado à criança em sofrimento psíquico, como o descompasso entre formação e prática, e a alienação ao saber de outros profissionais^(13)^. Para suprir essa lacuna, é preciso que o enfermeiro adote um referencial teórico para embasar a relação terapêutica, o que permite um cuidado autônomo e transformador^(14)^.

Nesse contexto, ao atuar com crianças em sofrimento psíquico, o BTD surge como alternativa de cuidado exclusivo do enfermeiro, e para leitura do que é apresentado pela criança nas sessões, é fundamental a adoção de um referencial teórico^(8,14)^. A psicanálise surge como possibilidade de leitura do brincar durante o BTD, ao tomá-lo como linguagem estruturada tal como o inconsciente, caracterizada pela articulação significante^(9)^. Ou seja, é preciso considerar que o inconsciente está estruturado como uma linguagem e que esta se dá por meio de significantes^(7,9)^. Assim, o brincar, além de linguagem de acesso ao inconsciente, também é uma prática significante que tem como objeto o brinquedo^(7,9)^.

Para sistematizar a implementação do BTD como intervenção, toma-se a opção de utilizar o processo de enfermagem (PE) como uma possível estratégia de cuidado que favorece ao enfermeiro assumir uma posição autônoma como agente terapêutico na relação enfermeiro-paciente^(13,14)^.

Assim, este estudo justifica-se pelas crescentes taxas de sofrimento psíquico na população infantojuvenil e alta demanda de atenção com tratamento intensivo, sendo os serviços comunitários de difícil acesso e segmentados; pela escassez de estudos que versam sobre o processo de cuidar do enfermeiro à criança em sofrimento psíquico, o que pode demonstrar uma lacuna de conhecimento no cuidado de enfermagem, inclusive no que se refere à utilização do BTD; e pela importância de enfermeiros estarem envolvidos nesse cuidado, ancorando-se em uma perspectiva teórica para implementação da assistência^(2,4,8,9,13)^.

## OBJETIVOS

Desenvolver uma proposta teórica sobre o processo de cuidado de enfermagem ao público infantojuvenil em sofrimento psíquico, considerando a estruturação do sujeito do inconsciente para o estabelecimento da relação enfermeiro-paciente por meio do BTD.

## MÉTODOS

Este artigo é produto da dissertação de mestrado “Cuidado à criança com transtorno mental por meio do Brinquedo Terapêutico Dramático: abordagem pela relação intersubjetiva”^(15)^, depositada no repositório institucional da universidade pública em que foi defendida, com acesso pelo *link:* https://hdl.handle.net/20.500.12733/9773.

### Referencial teórico-metodológico

Utilizou-se como referencial teórico e metodológico a psicanálise, que atua no campo da linguagem, em que a função da fala sob transferência, entendida como a repetição das formas de satisfação utilizadas para inaugurar relações, é compreendida por meio da leitura da repetição transferencial que apreende a linguagem do inconsciente, pois ele se expressa na lógica significante^(16,17)^.

Logo, considerar o inconsciente é trabalhar com significantes, inseridos na linguagem e conceituados como unidades fonemáticas que permitem que uma palavra possa adquirir múltiplos significados, visto que são manifestações inconscientes e, portanto, devem obedecer aspectos estruturais que se caracterizam pela: repetição; gravitação significante (quando uma palavra assume sentido singular para o sujeito para além do sentido pactuado na linguagem comum, o que favorece um novo traçado para a posição subjetiva); e direção (determina ao sujeito caminhos preferenciais para seus modos de relação com o Outro)^(7,18)^. Vale ressaltar que um significante não possui um único significado, mas sim carrega efeitos de significação que são inestimáveis^(7,18,19)^.

O saber do inconsciente possui furos, e neste trabalho, o furo no saber se caracterizou pela dificuldade da equipe de enfermagem em estabelecer seu papel e funções no cuidado de crianças em sofrimento psíquico, tendo como efeito um cuidado sem embasamento^(13)^.

Tomou-se como referencial teórico para elaboração do processo de cuidar o BTD^(12)^, e para articulação junto ao referencial teórico da psicanálise, tomou-se a leitura do inconsciente a partir de Freud^(20)^, Lacan^(18)^ e Ricardo Rodulfo^(7)^. Por meio da linguagem, o sujeito percebe sua existência e nomeia seu sofrimento psíquico, porém, no contexto infantil, a criança carece do domínio dessa^(7,17)^. O brincar surge como sua forma de comunicação, pois possibilita a ela expressar-se e, consequentemente, o acesso ao inconsciente^(7)^.

Apesar de alguns autores defenderem que há uma diferença entre o brincar e o brinquedo, é preciso salientar que, na língua portuguesa, “brincar”, “brincadeira” e “brinquedo” podem ou não ser sinônimos: enquanto os dois primeiros sempre se referem ao ato de brincar, o terceiro pode referir-se tanto à ação quanto ao objeto^(8)^. Neste contexto, quando nos referimos ao BT, estamos falando do ato de brincar, sendo o brinquedo (material) um facilitador, e não é pela ausência dele que a criança deixa de brincar, pois quando não há brinquedos à disposição, ela cria, usa a imaginação e brinca^(8,12)^.

Ao adotar o referencial psicanalítico, é preciso fazer alterações e ajustes teóricos nas etapas do BTD para que a emersão do sujeito do inconsciente seja atingida^(17,18,20)^. Assim, as etapas da sessão de BTD^(12)^ foram transpostas às etapas do PE, sendo trabalhadas da seguinte maneira: Estabelecendo relação; Explorando; Brincando; e Encerrando a sessão.

### Tipo de estudo

Trata-se de pesquisa teórica qualitativa, que consiste na construção de conexões e relações entre conceitos derivados de teorias prévias para desenvolvimento de um novo estudo^(21)^.

### Procedimentos metodológicos

O trabalho foi dividido em três etapas. A primeira consistiu em revisão integrativa acerca do cuidado de enfermagem desenvolvido com crianças em sofrimento psíquico, a qual possibilitou a apreensão dos conceitos significantes (que se repetem) sobre este cuidado: modelos de atenção à saúde, família, diagnóstico e manejo.

A segunda etapa pretendeu compreender o referencial teórico do brincar como linguagem do inconsciente para leitura da articulação significante^(7)^, o que permitiu, por meio da leitura uniformemente suspensa, assimilar os conceitos psicanalíticos de criança, significante, mito familiar, entrevistas preliminares e brincar.

Por fim, a terceira e última etapa foi realizada por meio da articulação entre os conceitos significantes do cuidado de enfermagem à criança em sofrimento psíquico e o referencial teórico do brincar como linguagem do inconsciente para a leitura da articulação significante^(7)^, de modo que modelos de cuidado em saúde foram articulados à criança e ao significante; família ao mito familiar; diagnóstico às entrevistas preliminares; e manejo ao brincar.

### Análise dos dados

Os dados foram analisados de acordo com a relação de transferência entre o pesquisador e o conteúdo investigado, na medida em que as leituras o tocaram para além da racionalidade adotada no estudo dos textos^(22,23)^. Isso ocorre porque não há apenas racionalidade na leitura para compreender uma articulação teórica, mas também processos inconscientes^(22,23)^.

Utilizaram-se as técnicas de associação livre e atenção uniformemente suspensa, que acontecem na perpétua revisão dos conceitos psicanalíticos, permitindo que o pesquisador mantenha-se alerta ao que possa aparecer ao não adotar uma postura categórica em que sua atenção seria direcionada apenas às ideias que confirmem o que já está estabelecido^(22-24)^. Sendo assim, o pesquisador se empenhou para não engessar a teoria, o que o permitiu se deparar com um desenvolvimento conceitual não totalizante^(23)^.

## RESULTADOS

Os resultados são apresentados em quatro quadros que compreendem cada etapa do BTD e seus respectivos suporte teórico (conceitos necessários para desenvolvimento das etapas), fases do PE, e atitudes e ações do enfermeiro (Quadro 1, Quadro 2, Quadro 3 e Quadro 4). Vale destacar que os quadros contêm orientações teóricas universais que devem ser consideradas em cada caso, na tentativa de singularizar a assistência de enfermagem ao reconhecer o que cada criança precisa em cada etapa do PE.

**Quadro 1:** Suporte teórico, fases do processo de enfermagem, e atitudes e ações do enfermeiro na etapa “Estabelecendo relação” do brinquedo terapêutico dramático

Etapa do brinquedo terapêutico dramático	Estabelecendo relação			
**Suporte teórico**	Modelo de atenção biopsicossocial: cuidado integral com respeito à singularidade	Registro infantil		Significantes
	Associação livre	Transferência		Mito familiar
	Pedido de cura endereçado ao profissional	Histerização: transformar o sintoma em enigma		Cuidado centrado na família: inclusão dos pais no tratamento
	Entrevistas preliminares: funções transferencial e sintomal		Endereçar-se à criança: há sofrimento?	
**Fases do processo de enfermagem**	Avaliação de enfermagem			
**Atitudes e ações do enfermeiro**	Considerar o registro infantil, associado à constituição do sujeito do inconsciente, para que o cuidado aconteça tendo em vista a singularidade, o que possibilita a integralidade defendida no modelo de atenção biopsicossocial	Apresentar-se, convidar a brincar e combinar os princípios que vão conformar as sessões (dia, horário, duração e local)		Permitir o expressar-se subjetivamente para além da posição ocupada no mito familiar, descobrindo se há sofrimento
	Proporcionar o brincar livre e o “tudo pode servir” para surgimento da associação livre	Incluir os pais no tratamento e fornece-lhes escuta, a fim de descobrir os principais traços do mito familiar e o lugar ocupado pela criança		Estabelecer a relação transferencial nas entrevistas preliminares, permitindo o endereçamento de uma demanda a ser transformada em sintoma e este em enigma

**Quadro 2:** Suporte teórico, fases do processo de enfermagem, e atitudes e ações do enfermeiro na etapa “Explorando” do brinquedo terapêutico dramático

Etapa do brinquedo terapêutico dramático	Explorando		
**Suporte teórico**	Caixa de brinquedos do brinquedo terapêutico dramático	Mito familiar: o Outro como corpo materno e tesouro de significantes	Entrevistas preliminares: funções transferencial, sintomal e diagnóstica
	Repetição significante	*Acting out*	Entrada no tratamento
	Espontaneidade do brincar	Complexo de Édipo	Esburacar
	Os destinos da criança: falo, sintoma ou fantasma		
**Fases do processo de enfermagem**	Avaliação de enfermagem	Diagnóstico de enfermagem	Planejamento de enfermagem
**Atitudes e ações do enfermeiro**	Permitir a exploração dos brinquedos e repetição da brincadeira, para que encene os significantes herdados do Outro enquanto mito familiar	Reconhecer os significantes para possibilitar o encontro de novos ou recolocar sua relação com eles, desobstruindo uma cadeia de significante	Identificar se há espontaneidade do brincar e estimular a recuperação de sua capacidade brincante, para que não haja apenas desejo familiar
	Atuar em conjunto com os pais para identificar os mitos familiares, o lugar ocupado pela criança na família e no desejo do Outro	Permitir o esburacar, perder o respeito ao brinquedo como condição para ser	Transformar o sintoma em enigma e traçar hipóteses sobre o diagnóstico estrutural
	Permitir o brincar como *acting out* mediador do cuidado		

**Quadro 3:** Suporte teórico, fases do processo de enfermagem, e atitudes e ações do enfermeiro na etapa “Brincando” do brinquedo terapêutico dramático

Etapa do brinquedo terapêutico dramático	Brincando		
**Suporte teórico**	O brincar como meio de comunicação	Aquisição da noção de volume: continente/conteúdo	Relação de objeto e linguagem: “fort/da”
	Transferência	Inclusões recíprocas	Desmame
	Formação de superfícies contínuas	Simbolização do desaparecimento	Idade para participar do brinquedo terapêutico dramático
	Constituição libidinal e estruturação do corpo		
**Fases do processo de enfermagem**	Avaliação de enfermagem	Diagnóstico de enfermagem	Planejamento de enfermagem
	Implementação de enfermagem		
**Atitudes e ações do enfermeiro**	Utilizar a transferência para estabelecer a comunicação por meio do brincar	Proporcionar a constância do *setting* e permitir a formação de continuidade com o ambiente para que inscreva-se sob a forma de uma superfície e consiga realizar operações de dentro/fora	Possibilitar brincadeiras que permitam simbolizar o desaparecimento do Outro, para que a separação não se torne sinônimo de destruição corporal
	Considerar os elementos que se apega como essenciais para constituição do sujeito em sua corporalidade libidinal	Receber as brincadeiras de relação continente/conteúdo para que elabore um tubo tridimensional e adquira a noção de volume	Aplicar o brinquedo terapêutico dramático como operador lógico para auxiliar no estabelecimento das funções do brincar, contribuindo para a constituição psíquica e estruturação corporal
	Identificar quais dificuldades lógicas a criança tenta resolver por meio do brincar e fornecer recursos para que ela atinja o desenvolvimento psíquico que encontra-se defasado		

**Quadro 4:** Suporte teórico, fases do processo de enfermagem, e atitudes e ações do enfermeiro na etapa “Encerrando a sessão” do brinquedo terapêutico dramático

Etapa do brinquedo terapêutico dramático	Encerrando a sessão	
**Suporte teórico**	*Setting* analítico	Tempo lógico
	Subjetividade	Constância do *setting*
**Fases do processo de enfermagem**	Evolução de enfermagem	
**Atitudes e ações do enfermeiro**	Estabelecer um contrato de continuidade respeitando a singularidade, considerando a particularidade de cada caso e possibilitando a emersão do inconsciente	Manter a constância do *setting* respeitando o contrato referente ao dia, horário, duração e local das sessões de brinquedo terapêutico dramático, o que auxilia na construção de superfícies contínuas
	Considerar as reações da criança diante do fim do brincar e construir intervenções que contribuam para que ela elabore a separação	Considerar o tempo lógico e estar ciente que o brincar pode se estender por mais de uma sessão

## DISCUSSÃO

A utilização do referencial teórico da psicanálise para adoção do brincar como intervenção pode permitir que o enfermeiro realize um cuidado contínuo e construído na relação terapêutica enfermeiro-paciente durante o BTD, o que possibilita o desenvolvimento das etapas do PE^(7,12,13)^. Para tal, é preciso que o enfermeiro considere as etapas de desenvolvimento do BTD, bem como o suporte teórico, e as atitudes e ações a serem utilizadas pelo profissional.

### Estabelecendo relação

Nesta etapa, o enfermeiro utiliza a transferência, compreendida como uma das funções das entrevistas preliminares, pois permite a apreensão da lógica do inconsciente^(16,25)^. A relação transferencial se inicia com a apresentação pessoal, o convite ao brincar e o estabelecimento dos princípios que vão nortear a sessão de BTD, devendo levar em conta a subjetividade^(12,17)^. Ao se apresentar, a criança coloca o enfermeiro em um lugar conforme a transferência, resultado da repetição das formas de satisfação utilizadas para responder à introdução de novos relacionamentos e que possui estreita relação com as experiências vivenciadas em seus primeiros anos^(16)^.

É por meio da relação transferencial que o enfermeiro adota como atitude a aplicação da função sintomal das entrevistas preliminares, ou seja, a criança lhe dirige uma demanda, respondida por meio de um sintoma causador de sofrimento psíquico, devendo tal demanda ser transformada em uma pergunta, o que entende-se como processo de histerização^(7,25,26)^. Desta forma, o brincar sob relação transferencial possibilita ao enfermeiro escutar o inconsciente da criança, permitindo que os significantes que marcaram sua constituição psíquica se manifestem durante as sessões^(27)^.

Também é por meio da transferência que surge a associação livre, aqui considerada como brincar livre^(24,27,28)^. É por meio do brincar que o enfermeiro estabelece uma comunicação com a criança, visto que ela ainda não detém o domínio total da linguagem; e a partir disso, ela endereça um pedido de cura ao profissional^(7,8)^. Permitir o brincar como meio de comunicação é extremamente importante, pois é no âmbito da linguagem que a função da fala sob transferência coloca em ato a realidade do inconsciente^(7-9,12,16,17)^.

Logo, é importante que o brincar siga os princípios da associação livre, pois deve ser espontâneo, sem ser dirigido, proporcionando a liberdade de escolha, a expressão de sentimentos e o aparecimento da subjetividade^(7,8,27)^. O enfermeiro deve assumir a ação de oferecer os brinquedos sem direcionamento, e também não deve direcionar o brincar, participando dele apenas quando solicitado^(8)^. O brincar livre adquire importância pois, sendo a criança quem traz os significantes, é somente ela que tem condições de produzir novas possibilidades para sua vida^(8,29)^. Outra característica do brincar livre é de que “tudo pode servir”^(7)^. Ou seja, a criança brinca quando toma qualquer objeto, constituindo uma formulação teórica de transição do acidental em necessário e estrutural, visto que ela compõe seu ego corporal com este tipo de material^(7)^.

Por meio da relação transferencial, o enfermeiro pode delinear hipóteses sobre o lugar ocupado pela criança no mito familiar, constituído por um tesouro de significantes e entendido como um repertório de linguagem que ela utilizará como base para suas relações futuras^(3,7)^. Para acessar a estrutura significante da criança e sua família, é importante que o enfermeiro atue de maneira a oferecer escuta aos pais, o que permite reconhecer os significantes que se repetem em seus discursos^(9,27)^.

Essa escuta pode ser pontual ou contínua, em conjunto com a criança ou não, porém levando em consideração que a inclusão e participação efetiva de familiares na sessão de BTD só podem acontecer se solicitadas e/ou autorizadas por ela^(7,8)^. Contudo, é importante que o discurso dos pais/familiares seja recebido no patamar do tratamento da criança, permitindo a ela que se expresse subjetivamente para identificar se há sofrimento^(27)^. Desta forma, abre-se a possibilidade de que ela, sob transferência, constitua sua posição subjetiva, deixando de ser objeto passivo do fantasma parental e exercendo papel ativo em seu próprio desejo^(27)^.

Essa maneira de atuar permite ao enfermeiro tomar a criança como efeito do significante, levando em conta seu sofrimento psíquico com respeito à sua singularidade, o que pode possibilitar o cuidado integral adotado no modelo de atenção biopsicossocial^(5,7,27)^. Para isso, é preciso considerar o registro infantil, o qual está associado à constituição do sujeito do inconsciente^(18,20,27)^. Sendo assim, na primeira etapa do BTD, o enfermeiro assume a posição de agente terapêutico e realiza a avaliação de enfermagem.

### Explorando

Nesta etapa, compõe-se uma caixa de brinquedos que será entregue à criança para que ela explore livremente^(12,30)^. Sugere-se que nela haja: objetos do cotidiano, como alimentos de plástico, móveis em miniatura e carrinho; figuras representativas, como bonecos (simulando familiares, profissionais de saúde, animais); materiais que estimulem a criatividade, como lápis coloridos, papel e massa de modelar; e brinquedos que permitam a manifestação de sentimentos, como chupeta, mamadeira, armas, tanques de guerra e soldados^(8,30)^. É quando ela entra em contato com esta caixa, composta por uma variedade de brinquedos, que a exploração se faz presente^(12)^.

O enfermeiro precisa se atentar a uma das principais funções do brincar como prática terapêutica: que ele se reproduza incessantemente, o que configura uma repetição significante^(7,31)^. Deve-se receber essa insistência, pois o que é repetido tende à elaboração simbólica pelo próprio movimento de repetição, que deve ser lido como um nó a decifrar^(7,9,31)^.

Tal repetição abre caminho à atuação (*acting out*) quando, por mecanismos de resistência, a criança é impossibilitada de recordar o que reprimiu, apresentando-o em ato^(31-33)^. A partir disso, coloca o enfermeiro em sua repetição, e este, ao identificá-la, deve interrogar a criança na relação transferencial, auxiliando-a a se expressar pela palavra, não pela ação^(17,31-33)^.

A repetição do brincar tem relação com o conceito de significante, quando a criança encena a apropriação dos significantes do Outro, os quais indicam a posição que ela é colocada no mito familiar e seu lugar ocupado no desejo deste Outro: como falo (marcada pelo desejo); sintoma (presa a um determinado traço da construção familiar em que não é possível inscrever uma diferença); ou fantasma (quando a função paterna fica apagada)^(7)^.

Além disso, possibilita ao profissional elaborar hipóteses sobre a posição da criança na linguagem por meio dos modos de negação do complexo de Édipo, que aborda as relações triangulares entre pais e criança, podendo ela direcionar sentimentos intensos como ódio e amor às figuras parentais^(7,18,34)^. Como resultado, é possível identificar as estruturas neurose, psicose ou perversão, o que permite ao enfermeiro exercer a função diagnóstica das entrevistas preliminares^(7,25,27)^. As funções transferencial e sintomal, iniciadas na etapa anterior, também estão presentes nesta e sinalizam a entrada no tratamento^(7,17,25)^.

O sintoma da criança pode se apresentar como uma resposta à problemática familiar, sendo preciso continuar o que se iniciou na etapa anterior: a inclusão dos pais no tratamento. Ao nascer, o bebê necessita de um intérprete, entendido como Outro ou função materna, que nomeará seus atos e, na medida em que interpreta seu estado e busca significações para apaziguá-lo, ele é inserido na lógica da linguagem, representada pelos significantes a ele oferecidos^(9,35)^. Ou seja, este Outro pode ser lido como corpo materno e tesouro de significantes^(7)^. Dessa forma, é essencial identificar os significantes do mito familiar, delimitar as posições de cada membro na família e reconhecer se a criança é marcada pelo novo e diferente^(3,7,27)^.

Como resultado, nas sessões de BTD, a criança busca significantes que a representam, expressa desejo de transformar os que já a representam, ou ainda, mais frequentemente, busca se desfazer de alguns deles^(7)^. O enfermeiro, neste caso, tem como ação favorecer condições para que ela encontre um significante ou recoloque sua relação com ele.

Nesta etapa, também é necessário detectar a presença ou ausência de espontaneidade no brincar, pois a criança pode estar brincando para satisfazer demandas que ela assume no adulto^(7)^. Caso a criança não consiga brincar espontaneamente, o enfermeiro deve auxiliar na recuperação ou desenvolvimento de sua capacidade brincante^(36)^.

A última atitude a ser observada nesta etapa é a permissão do brincar enquanto esburacar, pois produzir um furo é condição para ser^(7)^. As atividades mais intensas e regulares no primeiro ano de vida estão associadas à produção de buracos no corpo do Outro, momento em que o bebê retira e se apropria de significantes para constituir-se enquanto sujeito desejante, separando-se e diferenciando-se do corpo deste Outro^(7,37)^. Neste contexto, o enfermeiro deve permitir à criança perder o respeito ao brinquedo (objeto) como forma de esburacar e retirar significantes^(7)^.

Assim, na etapa “Explorando”, o enfermeiro dá continuidade à avaliação, elabora os diagnósticos e inicia o planejamento de enfermagem.

### Brincando

Nesta etapa, a criança realmente brinca, ou seja, comunica-se com o enfermeiro. Para isso, cria situações imaginárias que possibilitam a ela expressar e lidar com seu cotidiano, angústias, medos, tensões e conflitos, um modo de verbalizar seus afetos e suas experiências de vida^(7,12)^.

O brincar tem como primeira função, especialmente no primeiro ano de vida, a constituição libidinal e estruturação do corpo, isto é, assemelha-se ao processo de extrair materiais do corpo do Outro para fabricar o próprio corpo^(7,37)^. A criança utiliza os materiais extraídos em sequência: extrair-fabricar superfícies contínuas^(7)^.

Assim, o reencontro constante com o Outro perfurável possibilita ao bebê alcançar sua integração, isto é, permite encontrar matéria-prima para construção de superfícies contínuas^(7)^. É imprescindível que a criança se inscreva sob a forma de superfície para que seja capaz de realizar operações do tipo dentro/fora^(7)^.

Em pediatria, recomenda-se a criação de rotinas para o bebê, as quais nada mais são do que a fabricação de superfícies: o Outro deve fornecer, por meio delas, meios para construir uma cotidianidade^(7)^. É importante que o enfermeiro adote como ação o desenvolvimento do BTD em um ambiente protegido, com constância de *setting*, longe de oscilações excessivas da realidade externa, proporcionando a tranquilidade indispensável para a construção de superfícies^(7)^.

A segunda função do brincar se relaciona ao segundo momento na estruturação do corpo, ao qual se vê entregue um bebê: uma série de jogos de relação continente/conteúdo^(7)^. Para tal, é preciso considerar o conceito de inclusões recíprocas. Em outras palavras, a relação entre continente e conteúdo é reversível e ambígua, sendo um engano pressupor que há um conteúdo interno diferente de um continente externo^(7)^.

A partir da formação de superfícies contínuas e da elaboração de um tubo bidimensional, gradualmente a noção de volume pode ser alcançada em seu interior, o que permite o processo de diferenciação entre interno/externo e sujeito/objeto, tornando-o um tubo tridimensional^(7)^. Neste contexto, o enfermeiro, nas sessões de BTD, detém como atitude aceitar o brincar associado ao continente/conteúdo^(7)^, isto é, a repetição de brincadeiras de tirar e pôr objetos dentro de uma caixa, abrir e fechar armários e gavetas de forma desorganizada, por exemplo.

A terceira função do brincar surge por volta dos 9 meses, e manifesta-se por meio de jogos de esconde-esconde, práticas de desaparecimento e aparecimento^(7,20,31)^, resultando em brincadeiras mais elaboradas em que o gozo de ocultar-se é essencial^(7)^.

O desaparecimento, que até então não provocava nenhum prazer ou gerava angústia, torna-se um acontecimento libidinal, e a criança vê nele graça e pede repetição^(7)^. Uma das funções atribuíveis ao brincar, conhecida como “fort/da”, é, portanto, simbolizar o desaparecimento e dar representação à ausência^(7,20,31)^. Neste cenário, o desaparecimento do olhar do Outro já não causa fraturas desestruturantes nas superfícies contínuas, e a criança experiencia sua existência na ausência desse Outro^(7,31)^.

Esse modo de brincar está associado ao desmame, principalmente do olhar materno^(7)^. Esses breves momentos, quando a criança deixa cair o olhar que a sustenta, permitem o gozo duplicado do esconder-se e do reencontro^(7)^. Assim, a pergunta que a criança precisa direcionar, nos múltiplos brincares nas sessões de BTD, é: como pode existir algo ausente, que não é visível?

A conquista de uma operação depende da obtenção da anterior, isto é, destruições produzidas na superfície corporal prejudicarão a operação “fort/da”, pois a separação ficará implicada como sinônimo de destruição de si, de seu próprio corpo^(7)^.

Por esse motivo, uma das inovações deste estudo consiste em sugerir a ampliação da faixa etária de utilização do BTD, até então recomendada a crianças de 3 a 12 anos^(8)^, ao público infantojuvenil, ao considerar o brincar como um dispositivo simbólico que possibilita a emersão do discurso e, por consequência, o acesso ao inconsciente, como defende o referencial teórico da psicanálise^(7,16,18)^. É preciso que o enfermeiro considere a aplicação do BTD como operador lógico para auxiliar na implantação das funções que o brincar possui para a constituição psíquica, e seu uso não deve ser engessado em uma faixa etária determinada, tendo em vista que o sofrimento psíquico traz a precocidade ou o adiamento da constituição do sujeito^(7,27)^.

Logo nos primeiros meses de vida, já é possível observar que a criança responde às angústias de maneira somática, ou seja, com o corpo^(7)^. Neste contexto, o BTD pode ser aplicado com o início da estruturação simbólica da linguagem, a partir dos 6 meses, e por meio da leitura lógica e avaliação do enfermeiro caso a caso, pode se estender até quando o sujeito, seja criança ou adolescente, ainda precisa organizar a constituição de superfícies contínuas, a aquisição da noção de volume e a ressignificação das separações, visto que a falha na obtenção de tais funções pode causar repercussões para além dos 12 anos^(7,16)^.

É importante que o enfermeiro considere que a criança em idade para aplicação do BTD pode manifestar sintomas e necessidades que revelam que as funções estruturais do brincar foram falhas; neste caso, nas sessões, haverá a repetição de brincadeiras que demonstram o déficit no desenvolvimento destas^(7)^. Com base nisso, o profissional deve ofertar os recursos necessários, durante as sessões, para que ela alcance o desenvolvimento psíquico que se encontra defasado, considerando, para tal, a avaliação da lógica de sua constituição.

Com esse objetivo, o profissional adota como atitude receber as brincadeiras da criança e, a partir do que é expresso, questionar-se: quais dificuldades lógicas ela tem de se deparar, em determinados momentos, e que com seu brincar tenta resolver? A partir disso, a ação do enfermeiro é oferecer um cuidado que possibilite à criança uma forma de elaborar as questões apresentadas, por meio da etapa de implementação de enfermagem do PE.

É preciso considerar que, como esta etapa pode evidenciar novas descobertas sobre a criança e seu sintoma, é possível também que o enfermeiro formule outros diagnósticos de enfermagem e pense em novas intervenções a serem implementadas. Esse momento indica a íntima interligação entre as diversas etapas do PE.

### Encerrando a sessão

Por fim, nesta etapa, considerou-se a técnica psicanalítica do contrato com o paciente, que estabelece o *setting* analítico e define o tempo das sessões, sua frequência, valor e forma de pagamento^(25)^. Sobretudo, este contrato deve ser pensado no campo da ética, não das regras, isto é, deve ser elaborado em um ato psicanalítico, em que o profissional dá existência ao inconsciente, proporcionando o tratamento no particular de cada caso^(25)^.

Assim, o contrato das sessões de BTD deve dispor de condições, não regras impostas. Também é preciso levar em conta o tempo lógico, diferente do cronológico, pois o respeito não é pelo relógio, mas pelo tempo da relação significante^(38,39)^. Desta forma, o contrato acontece de maneira específica para cada criança, proporcionando a constância do *setting* e levando em conta as necessidades observadas pelo enfermeiro.

Aspectos como horários e frequência das sessões precisam ser combinados de acordo com a particularidade de cada caso, tendo o enfermeiro que assumir a ação de estipular condições para que o contrato das sessões de BTD seja benéfico para a criança. Além disso, o enfermeiro deve observar as diferentes reações da criança neste momento: aceita o fim da brincadeira, começa a brincar quando percebe o fim da sessão ou busca prolongá-la, o que indica que, para ela, o brincar ainda não terminou^(12)^?

Tais reações devem ser lidas pelo profissional com o objetivo de adequar o tempo de cada sessão à subjetividade da criança. Isto é, o enfermeiro emprega como atitude estipular um contrato indicando o horário de início e término da sessão; contudo, pode fazer alterações em sua duração enquanto ela acontece, entendendo que seu encerramento também é considerado uma ação.

Vale destacar que o fim da sessão não significa o término do brincar, pois o que se considera no referencial psicanalítico é o tempo lógico^(38)^. A criança brinca como se não houvesse intervalo entre as sessões, de modo que uma sessão é continuidade da anterior e quem decide o final é somente ela^(12)^.

Ao considerar o PE, nesta etapa, o enfermeiro realizará a evolução de enfermagem e registro, ou seja, avaliará como a criança responde às intervenções implementadas, e dará continuidade ao cuidado ou pensará novas estratégias mais adequadas ao tratamento^(13)^, que deverão ser registradas em prontuário.

### Limitações do estudo

A limitação deste estudo versa sobre a dificuldade em transmitir conceitos robustos de um referencial teórico psicanalítico ao enfermeiro que opta adotá-lo para desenvolver seu cuidado em saúde mental infantojuvenil. A formação em psicanálise acontece por meio do tripé análise pessoal, supervisão e estudo da teoria^(40)^, e a proposta aqui defendida sustenta dois destes pilares: além de ofertar cuidado às crianças em sofrimento psíquico, sua aplicação com supervisão, aliada ao estudo dos conceitos trabalhados e análise pessoal, constitui o próprio espaço de formação psicanalítica.

### Contribuições para a área da Enfermagem

Este estudo apresenta como avanço central para a área da enfermagem em saúde mental infantojuvenil uma possibilidade de cuidado contínuo, o qual é elaborado na relação enfermeiro-paciente durante a realização das etapas do BTD. Para desenvolvê-las, o enfermeiro deve desenvolver competências, ancorando-se em um suporte teórico para embasar o cuidar, aqui considerado o brincar e suas funções na constituição psíquica. A partir disso, é possível que construa seu cuidado norteado pelas etapas do PE.

## CONSIDERAÇÕES FINAIS

Este estudo respondeu ao objetivo de desenvolver uma proposta teórica sobre o processo de cuidado de enfermagem para o público infantojuvenil em sofrimento psíquico por meio do BTD, ao considerar a estruturação do sujeito do inconsciente para o estabelecimento da relação enfermeiro-paciente.

Para tal, o processo de cuidado de enfermagem aqui proposto considera o brincar como meio de comunicação da criança, a fim de desenvolver a relação terapêutica durante as etapas do BTD: Estabelecendo relação; Explorando; Brincando; e Encerrando a sessão. O cuidado elaborado nas etapas do PE acontece na medida em que o enfermeiro se alicerça em um suporte teórico que será necessário para fundamentar suas ações e atitudes diante da criança em sofrimento psíquico.

Ao embasar teoricamente ações e atitudes para desenvolvimento do PE, o enfermeiro que adota o referencial teórico psicanalítico pode aplicar o BTD como operador lógico para identificar e atuar em falhas na constituição de superfícies contínuas, na aquisição da noção de volume e na ressignificação das separações, o que pode auxiliar no desenvolvimento psíquico para sustentação da subjetividade e estruturação de um corpo que encaminhe a uma vivência autônoma e singular.

## Data Availability

Os dados da pesquisa estão disponíveis no corpo do artigo.
